# The Receptor Tyrosine Kinase c-Met Promotes Lipid Accumulation in 3T3-L1 Adipocytes

**DOI:** 10.3390/ijms24098086

**Published:** 2023-04-29

**Authors:** Yu-Kyoung Park, Byeong-Churl Jang

**Affiliations:** 1 Department of Molecular Medicine, College of Medicine, Keimyung University, 1095 Dalgubeoldaero, Dalseo-gu, Daegu 42601, Republic of Korea; westside1140@hanmail.net; 2 Department of Physiology, Senotherapy-Based Metabolic Disease Control Research Center, College of Medicine, Yeungnam University, 170, Hyeonchung-ro, Nam-gu, Daegu 42415, Republic of Korea

**Keywords:** c-Met, JNJ38877605, lipid accumulation, AMPK, 3T3-L1

## Abstract

The receptor tyrosine kinase c-Met is elaborated in embryogenesis, morphogenesis, metabolism, cell growth, and differentiation. JNJ38877605 (JNJ) is an inhibitor of c-Met with anti-tumor activity. The c-Met expression and its role in adipocyte differentiation are unknown. Here, we investigated the c-Met expression and phosphorylation, knockdown (KD) effects, and pharmacological inhibition of c-Met by JNJ on fat accumulation in murine preadipocyte 3T3-L1 cells. During 3T3-L1 preadipocyte differentiation, strikingly, c-Met expression at the protein and mRNA levels and the protein phosphorylation on Y1234/1235 and Y1349 is crucial for inducing its kinase catalytic activity and activating a docking site for signal transducers were increased in a time-dependent manner. Of note, JNJ treatment at 20 μM that strongly inhibits c-Met phosphorylation without altering its total expression resulted in less lipid accumulation and triglyceride (TG) content with no cytotoxicity. JNJ further reduced the expression of adipogenic regulators, including CCAAT/enhancer-binding protein-α (C/EBP-α), peroxisome proliferator-activated receptor-γ (PPAR-γ), fatty acid synthase (FAS), acetyl CoA carboxylase (ACC), and perilipin A. Moreover, JNJ treatment increased cAMP-activated protein kinase (AMPK) and liver kinase B-1 (LKB-1) phosphorylation but decreased ATP levels. Significantly, KD of c-Met suppressed fat accumulation and triglyceride (TG) quantity and reduced the expression of C/EBP-α, PPAR-γ, FAS, ACC, and perilipin A. Collectively, the present results demonstrate that c-Met is a novel, highly conserved mediator of adipogenesis regulating lipid accumulation in murine adipocytes.

## 1. Introduction

Obesity is a significant factor that gives rise to noncontagious diseases, particularly hyperlipidemia, type 2 diabetes, and cancer [1]. Increased calorific imbalance leads to adipocyte differentiation and a surplus of fat, largely in the manner of triglyceride (TG) accumulation, ultimately leading to obesity progression [2,3].

Adipocyte differentiation (adipogenesis) is the fibroblast-like morphology of the preadipocyte development process into adipocyte-like cells [4]. Adipogenesis is tightly controlled by the expressions and phosphorylations (activations) of several transcription factors such as CCAAT/enhancer-binding proteins (C/EBPs), peroxisome proliferator-activated receptors (PPARs) [5,6], and Janus-activated protein kinase (JAK)/signal transducer and activator of transcription (STAT) signaling complexes [7]. Furthermore, lipogenic enzymes, including fatty acid synthase (FAS) and acetyl CoA carboxylase (ACC), and lipid droplet (LD)-correlated proteins, such as perilipin A, are also essential for adipogenesis [8,9,10,11]. Moreover, the enzymic and metabolic regulators, namely cAMP-activated protein kinase (AMPK), protein kinase A (PKA), extracellular signal-regulated protein kinase-1/2 (ERK-1/2), and adenosine 3′,5′-cyclic monophosphate (cAMP) also has a role in regulating adipogenesis [9,12,13].

To explore the regulation of transcriptional circumstance in white preadipocyte differentiation and function, we have recently conducted RNA sequencing in differentiating 3T3-L1 mouse white preadipocytes from the preadipocytes (day 0 (D0)) to D2 and D7 of the early and late stages of cell differentiation and found that the expression levels of multiple RNAs including mesenchymal-epithelial transition factor (Met or c-Met) are increased by the time difference. Of interest, c-Met was reported to be a novel immensely upregulated during adipogenesis. c-Met, also called hepatocyte growth factor receptor (HGFR), is a receptor tyrosine kinase encoded by the *MET* proto-oncogene [14]. c-Met is an attractive anti-tumor therapy target due to its oncogenic/pro-survival/anti-apoptotic/pro-angiogenic role in cancer biology [15,16].

However, c-Met expression, phosphorylation, and its role in white preadipocyte differentiation remain undisclosed. Our study demonstrated the expression and c-Met function in 3T3-L1 cell adipogenesis using c-Met knockdown and pharmacological inhibition with JNJ38877605 (JNJ) [17].

## 2. Results

### 2.1. c-Met Expression and Phosphorylation Are Vastly Upregulated throughout 3T3-L1 Preadipocyte Differentiation

**Figure 1:**
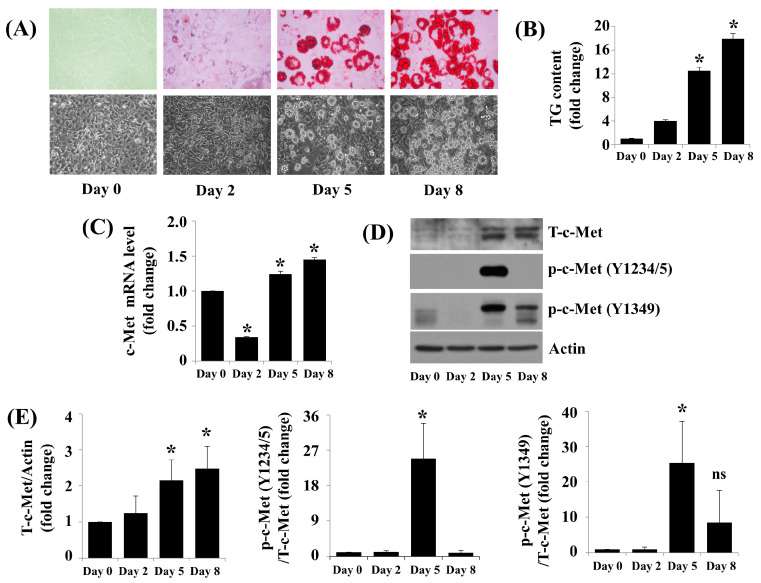
Fat accumulation and c-Met expression in protein and mRNA levels throughout 3T3-L1 cell differentiation. (**A**) 3T3-L1 cells were differentiated by growing them under an induction medium at day 0 (D0), D2, D5, and D8. At each time point, intracellular fat accumulation was assessed by Oil Red O staining (upper group images) and phase-contrast images (lower group images). (**B**) 3T3-L1 cells were differentiated by growing it under an induction medium at D0, D2, D5, and D8. At each time point, the intracellular TG content was quantified by AdipoRed assay. Values are mean ± SE of data from three independent experiments with three replicates. * *p* < 0.05 vs. control (D0). (**C**) 3T3-L1 cells were differentiated by growing them under an induction medium at D0, D2, D5, and D8. At each time point, total cellular RNA was obtained and examined by real-time qPCR analysis. Values are mean ± SE of data from three independent studies in three replicates. * *p* < 0.05 vs. control (D0). (**D**) 3T3-L1 cells were differentiated by growing it under an induction medium at D0, D2, D5, and D8. At each time point, whole-cell lysates were conceived and interpreted by Immunoblotting. (**E**) The densitometry data of immunoblotting analysis. * *p* < 0.05 vs. control. (**D**) in triplicate studies. NS, not significant.

We scrutinized cellular fat deposition and TG levels in day 0 (D0), D2, D5, and D8 of 3T3-L1 preadipocyte differentiation by utilizing Oil Red O staining and AdipoRed assays. There was a marked increase in fat deposition and TG levels in 3T3-L1 cells on D5 and D8 (Figure 1A,B) following the initiation of differentiation. c-Met transcripts were slightly decreased on D2 but induced on D5 and D8 compared with D0 3T3-L1 cell levels (Figure 1C). c-Met protein was greatly induced 3T3-L1 cell differentiation D5 and D8 (Figure 1D). c-Met is phosphorylated on tyrosine (Y) residues Y1234 and Y1235 (thereafter Y1234/5), inducing its kinase catalytic activity [18], and Y1349 and Y1356 in the C-terminus, initialize a docking site for signal transducers [19,20]. Strikingly, much higher phosphorylation levels of c-Met at Y1234/5 and Y1349 were detected in 3T3-L1 preadipocytes on D5 in opposition to D0, D2, or D8 (Figure 1D,E).

### 2.2. Pharmacological Inhibition of c-Met by 20 µM JNJ Suppresses Fat Deposition and Reduces TG Levels throughout 3T3-L1 Preadipocyte Differentiation in a Concentration- and Time-Dependent Manner

**Figure 2:**
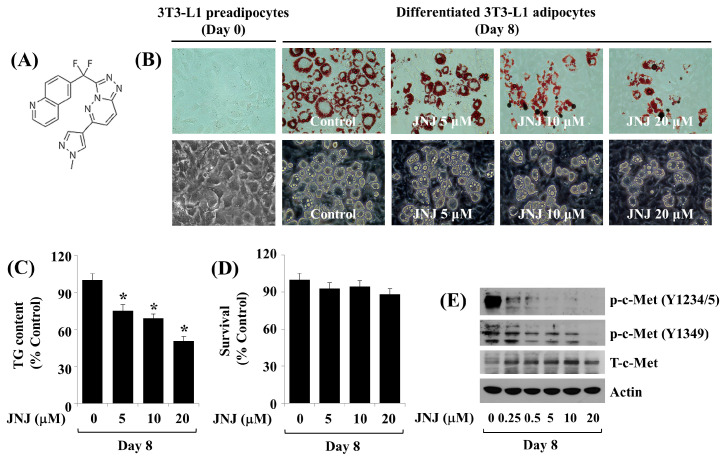
Effects of JNJ38877605 (JNJ) on fat accumulation, TG levels, cell growth, and c-Met expression and phosphorylation during 3T3-L1 cell differentiation. (**A**) The chemical structure of JNJ. (**B**) 3T3-L1 cells were differentiated using an induction medium with or without JNJ for 8 days. The intracellular fat accumulation was measured by Oil Red O staining (upper panels) and phase-contrast image (lower panels). (**C**) 3T3-L1 cells were differentiated using an induction medium with or without JNJ for 8 days. Intracellular TG content was examined by AdipoRed assay. Values are mean ± standard error (SE) of data from three independent studies with three replicates. * *p* < 0.05 vs. control. (**D**) 3T3-L1 cells were differentiated using an induction medium with or without JNJ for 8 days. The number that survived in control (DMSO, 0.1%) or JNJ-treated 3T3-L1 cells was calculated by cell count analysis. The cell count assay was established in triplicates. Data are mean ± SE of three independent experiments. * *p* < 0.05 vs. control. (**E**) 3T3-L1 preadipocyte differentiation induced by growing it under an induction medium with or without JNJ at the designated doses for 8 days. Whole-cell lysates and total RNA were collected and evaluated by immunoblotting and qPCR subsequently. * *p* < 0.05 vs. control.

Next, we evaluated the effect of JNJ (Figure 2A), an inhibitor of c-Met, at various concentrations (5, 10, and 20 µM) on the fat accumulation, TG levels, and cell growth on day 8 (D8) of 3T3-L1 preadipocyte differentiation. When compared with the vehicle-treated cells, treatment with JNJ caused a concentration-dependent reduction in fat accumulation (Figure 2B) and TG content (Figure 2C). JNJ up to 20 µM was not cytotoxic to 3T3-L1 cells (Figure 2D). We next examined the efficacy of JNJ in inhibiting the phosphorylation of c-Met on Y1234/5 and Y1349 on D8 of 3T3-L1 preadipocyte differentiation. Even JNJ at 0.25 µM greatly inhibited c-Met Y1234/5 phosphorylation (Figure 2E). In addition, JNJ at 0.5 µM was able to suppress c-Met Y1349 phosphorylation partially. Maximal inhibitory effects on c-Met Y1234/5 and Y1349 phosphorylation were at 20 µM JNJ. Because JNJ at 20 µM most strongly inhibited fat accumulation and reduced TG levels, this concentration was designated for further studies.

### 2.3. JNJ (20 µM) Greatly Inhibits c-Met Y1234/5 and Y1349 Phosphorylation throughout 3T3-L1 Preadipocyte Differentiation

**Figure 3:**
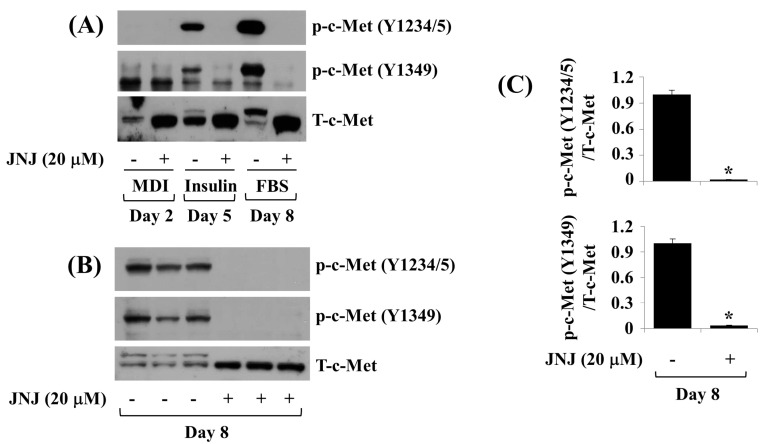
Effects of JNJ38877605 (JNJ) on c-Met expression and phosphorylation throughout 3T3-L1 preadipocyte differentiation. (**A**) 3T3-L1 cells were differentiated into adipocytes by growing in an induction medium with or without JNJ (20 µM) on days 2, 5, and 8. At each time point, whole-cell lysates were prepared and subjected to immunoblotting. (**B**) 3T3-L1 cells were differentiated into adipocytes by growing in an induction medium with or without JNJ (20 µM) only for 8 days. Whole-cell lysates were prepared and subjected to immunoblotting. Immunoblotting analysis in triplicate studies on day 8. (**C**) The densitometry data of (**B**). * *p* < 0.05 in opposition to JNJ-free control level at the designated day.

Next, we scrutinized the effect of JNJ (20 µM) on c-Met Y1234/5 and Y1349 phosphorylation throughout 3T3-L1 preadipocyte differentiation over time. Treatment by 20 µM JNJ strongly repressed c-Met Y1234/5 and Y1349 phosphorylation on D5 and D8. Triplicate experiments confirmed the capability of JNJ to almost completely inhibit c-Met Y1234/5 and Y1349 phosphorylation on D8 of 3T3-L1 preadipocyte differentiation (Figure 3B,C).

### 2.4. JNJ (20 µM) Downregulates the Expression of Adipogenic Transcription Factors (C/EBP-α and PPAR-γ) throughout 3T3-L1 Preadipocyte Differentiation

**Figure 4:**
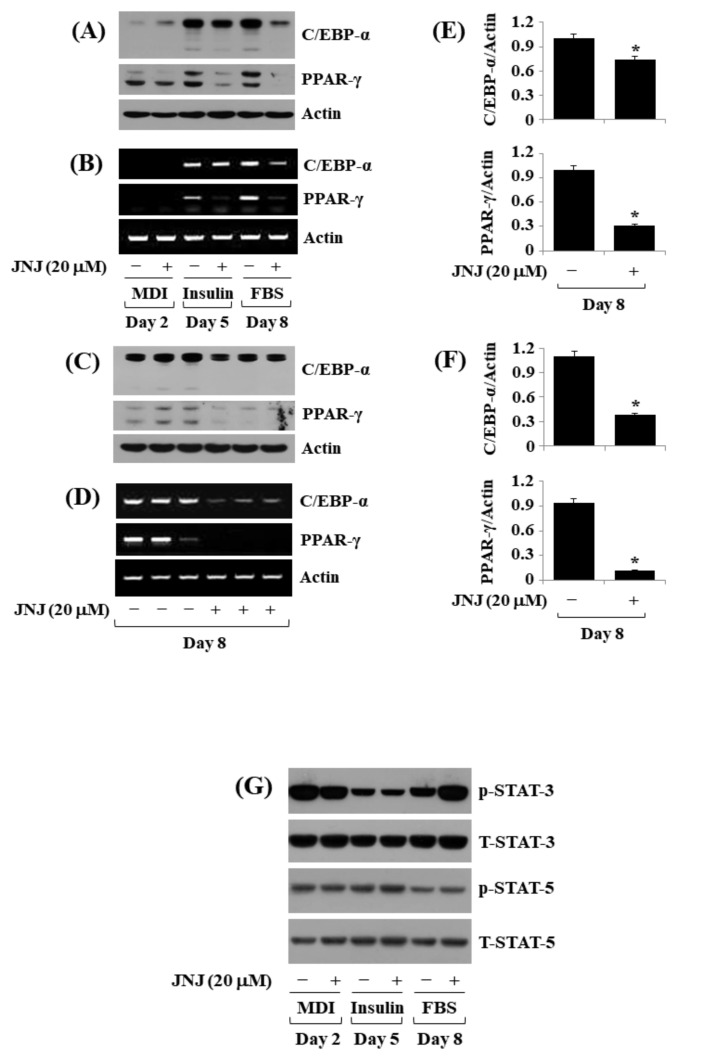
Effect of JNJ38877605 (JNJ) on the expression and phosphorylation levels of C/EBP-α, PPAR-γ, and STAT-3/5 throughout 3T3-L1 preadipocyte differentiation. (**A**) 3T3-L1 cells were differentiated into adipocytes by growing in an induction medium with or without JNJ (20 µM) on days 2, 5, and 8. At each time point, whole-cell lysates were prepared and evaluated using immunoblotting. (**B**) Total cellular RNA from the same conditioned cells as (**A**) at each time point was extracted and evaluated using RT-PCR analysis. (**C**) Immunoblotting analysis in triplicate experiments on day 8. (**D**) RT-PCR analysis in triplicate experiments on day 8. (**E**,**F**) The densitometry data of (**C**,**D**). (**G**) Whole-cell lysates from the same conditioned cells as (**A**) at each time point was collected and evaluated using immunoblotting.* *p* < 0.05 compared to JNJ-free control level at the designated day.

Next, we tested the effect of JNJ (20 µM) on the expression and phosphorylation levels of C/EBP-α, PPAR-γ, and STAT-3/5, known adipogenic transcription factors, throughout 3T3-L1 preadipocyte differentiation over time. JNJ (20 µM) treatment caused the reduced expression of C/EBP-α and PPAR-γ at their protein and mRNA levels on D5 and D8 of the cell differentiation (Figure 4A,B); further confirmed in triplicate on D8 of the cell differentiation (Figure 4C–F). On the contrary, treatment with JNJ (20 µM) did not modulate the STAT-3/5 phosphorylation levels at times tested; rather it increased STAT-3 phosphorylation on D8 of the cell differentiation robustly (Figure 4G). STAT-3/5 total protein expression levels remained unaffected under these studies.

### 2.5. JNJ (20 µM) Downregulates FAS, Perilipin A, and Leptin Expressions throughout 3T3-L1 Preadipocyte Differentiation

**Figure 5:**
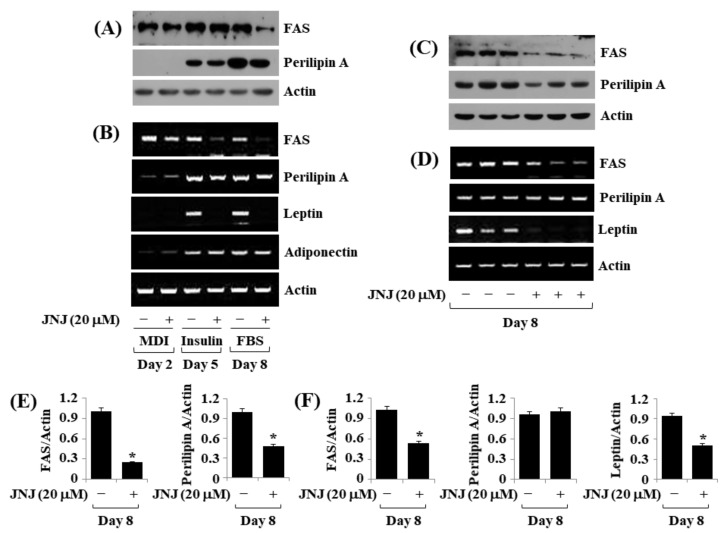
Effect of JNJ38877605 (JNJ) on the protein and mRNA expressions of FAS, perilipin A, leptin, and adiponectin during 3T3-L1 preadipocyte differentiation. (**A**) 3T3-L1 preadipocyte differentiation was induced by growing it under an induction medium with or without JNJ (20 µM) on day 2, 5, and 8. At each time point, whole-cell lysates were collected and evaluated by immunoblotting. (**B**) Total cellular RNA from the same conditioned cells as (**A**) at each time point was extracted and evaluated using RT-PCR analysis. (**C**) Immunoblotting analysis in triplicate on day 8. (**D**) RT-PCR analysis in triplicate on day 8. (**E**,**F**) The densitometry data of (**C**,**D**), subsequently. * *p* < 0.05 compared to the control group level (without JNJ) on the designated day.

Additionally, we tested the effect of JNJ (20 µM) on the expression levels of FAS and perilipin A throughout 3T3-L1 preadipocyte differentiation over time. JNJ treatment (20 µM) was shown to inhibit the protein expression of FAS and perilipin A (Figure 5A) as well as their mRNA expressions (Figure 5B) on D8 of 3T3-L1 cell differentiation. We further evaluated the effect of JNJ at 20 µM on the mRNA expression levels of leptin and adiponectin, well-known adipokines, throughout the cell differentiation. Distinctly, JNJ strongly inhibited leptin but not adiponectin, mRNA expression on D5 and D8 of 3T3-L1 preadipocyte differentiation. Triplicate experiments affirmed JNJ’s capability to inhibit FAS, perilipin A, and leptin expressions of D8 on cell differentiation (Figure 5C–F).

### 2.6. JNJ (20 µM) Alters the Phosphorylation and Expression Levels of AMPK, LKB-1, and ACC throughout 3T3-L1 Preadipocyte Differentiation

**Figure 6:**
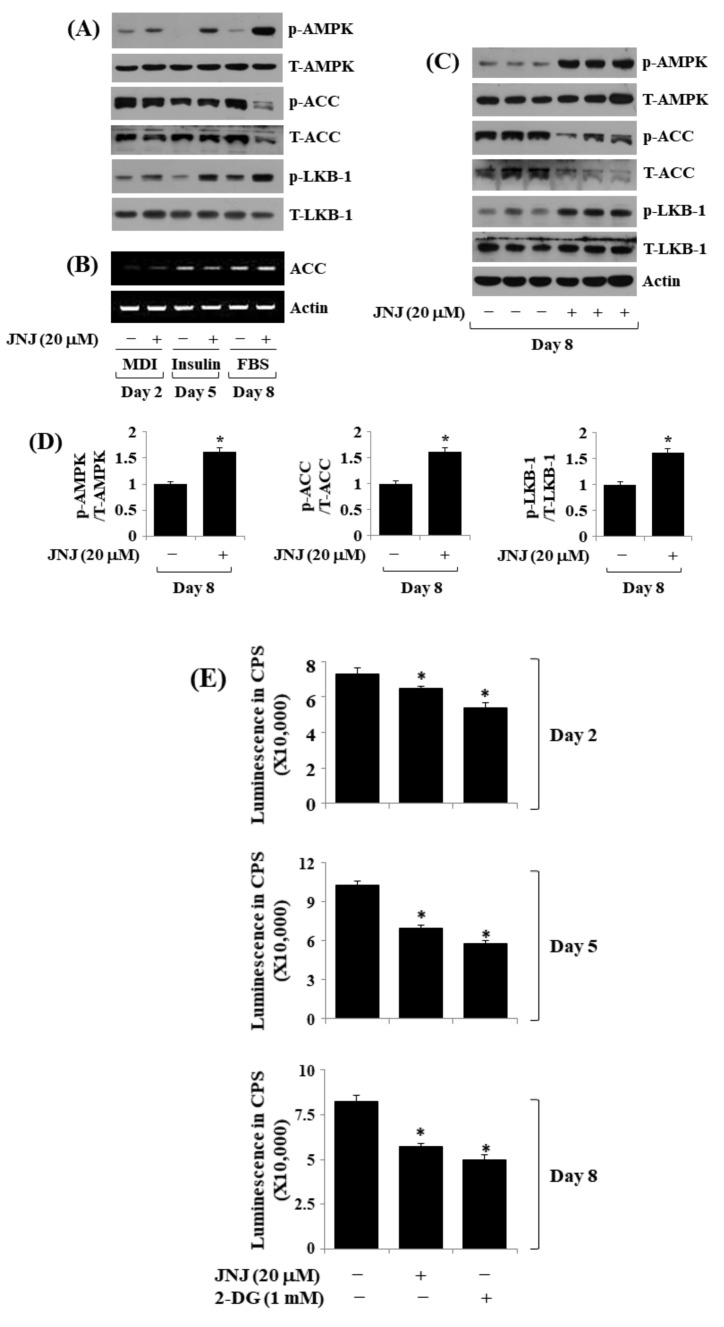
Effect of JNJ38877605 (JNJ) on the expression and phosphorylation of AMPK, ACC, and LKB-1 as well as ATP content during 3T3-L1 preadipocyte differentiation. (**A**) 3T3-L1 cells were induced by growing it under an induction medium with or without JNJ (20 µM) on days 2, 5, and 8. At each time point, whole-cell lysates were prepared and evaluated by immunoblotting. (**B**) Total cellular RNA from the same conditioned cells as (**A**) at each time point was extracted and evaluated using RT-PCR analysis. (**C**) Immunoblotting analysis in triplicate experiments on day 8. (**D**) The densitometry data of (**C**). * *p* < 0.05 compared to the value of JNJ free control on the designated day. (**E**) 3T3-L1 cells were induced by growing it under an induction medium with or without JNJ (20 µM) or 2-deoxyglucose (2-DG), a known ATP-reducing agent on day 2, 5, and 8, subsequently. At each time point, intracellular ATP levels were measured by an ATP measurement kit. * *p* < 0.05 compared to the control (without JNJ or 2-DG) value on designated day.

Next, we investigated the effect of JNJ (20 µM) on the expression and phosphorylation levels of AMPK and LKB-1 throughout 3T3-L1 preadipocyte differentiation over time. Notably, treatment with JNJ (20 µM) instigated a time-dependent surge in AMPK phosphorylation while maintaining total AMPK protein expression in D2, D5, and D8 of 3T3-L1 cell differentiation (Figure 6A). ACC is an AMPK downstream effector [21], whereas LKB-1 is an AMPK upstream activator [22] in adipogenesis of 3T3-L1 cells. Of interest, while JNJ treatment (20 µM) markedly reduced ACC phosphorylation and expression in D8, but not D2 and D5, of the cell differentiation, the same treatment caused a time-dependent increase in LKB-1 phosphorylation at times tested. In contrast, the JNJ treatment did not greatly affect ACC mRNA expression throughout 3T3-L1 preadipocyte differentiation (Figure 6B). Triplicate experiments further confirmed the JNJ’s capability in increasing AMPK and LKB-1 phosphorylation but decreasing ACC phosphorylation and expression on D8 of 3T3-L1 cell differentiation (Figure 6C,D). Considering that AMPK phosphorylation is also controlled by cellular AMP/ATP ratio adjustment [23], we then scrutinized the JNJ effect on cellular ATP levels throughout 3T3-L1 preadipocyte differentiation. 2-Deoxyglucose (2-DG), the non-metabolizable glucose which depletes cellular ATP content [24], was added as a control. Compared with 3T3-L1 cells at D0, treatment with JNJ (20 µM) significantly reduced ATP levels on D2, D5, and D8 of the cell differentiation, similar to 2-DG (Figure 6E).

### 2.7. Knockdown (KD) of c-Met Reduces the Fat Accumulation, TG Content, and Expression and Phosphorylation Levels of C/EBP-α, PPAR-γ, FAS, ACC, and Perilipin A during 3T3-L1 Preadipocyte Differentiation

**Figure 7:**
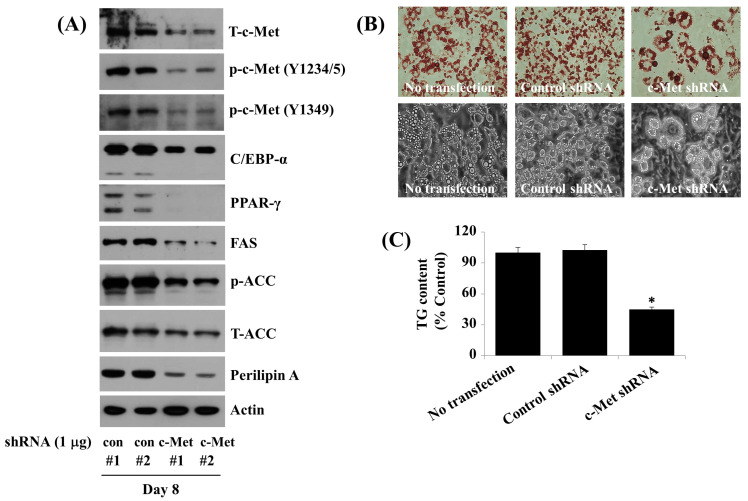
Effects of c-Met knockdown on the fat accumulation and TG content as well as the expression and phosphorylation of C/EBP-α, PPAR-γ, FAS, ACC, and perilipin A during3T3-L1 preadipocyte differentiation. (**A**) 3T3-L1 cells were transfected with two sets of control shRNA or c-Met shRNA for 48 h. Respective control (#1, #2) or c-Met (#1, #2) shRNA-transfected 3T3-L1 cells were differentiated by growing the cell under an induction medium for 8 days. Whole-cell lysates were prepared and evaluated using immunoblotting. (**B**) Intracellular fat accumulation in the same conditioned cells as (**A**) was examined by Oil Red O staining (upper group images) and phase-contrast image (lower group images). (**C**) Intracellular TG content in the same conditioned cells as (**A**) was measured by AdipoRed assay. Values are mean ± SE of data from three independent studies with three replicates. * *p* < 0.05 vs. control.

To directly see the role of c-Met in fat accumulation during 3T3-L1 preadipocyte differentiation, we established a stably pooled 3T3-L1 preadipocytes that were transfected with control or c-Met shRNA and selected in the presence of puromycin. Apparently, c-Met shRNA-transfected cells (Met #1 and 2) had significantly lower expression levels of endogenous c-Met on D8 of the cell differentiation D8 in opposition to control shRNA-transfected cells (con #1 and 2) (Figure 7A). Notably, there were also lower expression and phosphorylation levels of C/EBP-α, PPAR-γ, FAS, ACC, and perilipin A in c-Met shRNA-transfected 3T3-L1 cells on D8 of differentiation in opposition to control shRNA-transfected cells. Importantly, the knockdown of c-Met resulted in less fat accumulation and TG levels in 3T3-L1 cells on D8 of differentiation in opposition to control shRNA-transfected cells (Figure 7B,C).

## 3. Discussion

Evidence suggests that c-Met is pivotal in metabolic diseases, including obesity, insulin resistance, and type 2 diabetes [25]. Thus, c-Met and its inhibitor(s) could regulate obesity and related diseases. Currently, c-Met expression and its function during preadipocyte differentiation are still undetermined. Our study exhibited that c-Met is substantially expressed and phosphorylated during 3T3-L1 preadipocyte differentiation. Moreover, in this study, we provided experimental evidence that the upregulated c-Met plays a crucial role in fat accumulation during the adipocyte differentiation of 3T3-L1 preadipocytes, since gene silencing (KD) and pharmacological inhibition by JNJ cause less fat deposition and TG levels in 3T3-L1 preadipocyte differentiation. We also demonstrated that the lipid-lowering effect of c-Met KD or JNJ during 3T3-L1 preadipocyte differentiation is modulated via control of the expression and phosphorylation levels of C/EBP-α, PPAR-γ, FAS, ACC, perilipin A, AMPK, and LKB-1.

In the beginning, we illustrated that c-Met expression and phosphorylation are very low or not detectable in D0 and D2 of 3T3-L1 cell differentiation, but their levels are markedly elevated on D5 and D8. It is known that upon HGF binding, c-Met is phosphorylated on Y1234/5, which induces its kinase activity [18], and on Y1349/Y1356, which engages various docking and adapter proteins, such as Gab1 [26], and triggers the activation of multiple signal transducers, such as PI3K/PKB and Ras [27,28,29], and biological responses [16,20,30]. The HGF/c-Met activation contributes to cell growth, metabolism, angiogenesis, morphogenesis, and differentiation [16,31]. We found that the phosphorylation of c-Met on both Y1234/5 and Y1349 and its total protein and mRNA expression are time-dependently increased particularly on D5 and D8 of 3T3-L1 cell differentiation, suggesting that c-Met is active and may lead to a signaling cascade requiring an abundance of kinases and factors that elicits its biological activities (lipid accumulation herein).

It has been previously shown that capmatinib, a selective inhibitor of c-Met with anti-cancer activity, attenuates adipogenesis [32] and miR-206-3p, a microRNA to inhibit c-Met expression, blocks adipogenesis [33]. To date, the function of c-Met in adipocyte differentiation remains unclear. JNJ is an inhibitor of c-Met with anti-cancer activity [17,34], but its anti-obesity effect and mechanism is still undisclosed. In mice, the global knockout of c-Met is embryonically lethal [35,36], and no transgenic model of c-Met has addressed a role in adipogenesis [37]. To present c-Met as a novel white adipocyte differentiation regulator and reposition JNJ as an anti-obesity agent, we evaluated the JNJ effect on fat accumulation and TG levels throughout 3T3-L1 preadipocyte differentiation, also called adipogenesis. Strikingly, we observed that treatment with JNJ at 20 µM strongly reduced fat accumulation and TG levels throughout adipogenesis in 3T3-L1 cells without cytotoxicity, supporting the drug’s anti-adipogenic/anti-lipogenic effect. The present findings show that in addition to JNJ, INCB28060, another c-Met inhibitor, could suppress fat deposition and TG levels during 3T3-L1 cell differentiation with no cytotoxicity (Supplementary Figure S1) and a previous study suggested that capmatinib, another selective inhibitor of c-Met, attenuates adipogenesis [32]; it is believed that the ability to reduce lipid deposition in 3T3-L1 preadipocyte differentiation is not limited to JNJ but is a c-Met inhibitor class effect. Preadipocyte differentiation is regulated by the expression and phosphorylation of multiple adipogenesis-related transcription factors including C/EBP-α, PPAR-γ, and STAT-3/5 [7,38]. c-Met regulation of C/EBP-α, PPAR-γ, as well as STAT-3/5 was still undefined. In this study, JNJ strongly reduced the expression levels of C/EBP-α and PPAR-γ without altering the phosphorylation levels of STAT-3/5 in the 3T3-L1 preadipocyte differentiation process. FAS, a lipogenic enzyme [8], and perilipin A, a stabilizer of the newly formed LDs [10,11], support their involvement in preadipocyte differentiation. Because JNJ greatly lessens the expression levels of FAS and perilipin A during 3T3-L1 cell differentiation, it is likely that the JNJ’s lipid-lowering effect is associated with the downregulation of FAS and perilipin A. Adipose tissue produces a range of adipokines, including leptin and adiponectin [39,40]. Adiponectin levels reduce in increased adiposity conditions, and those of leptin increase in obesity [41,42]. Adiponectin is involved in the lowering of blood glucose and lipids, preserving insulin to remain sensitive, as well as expressed highly in the oxidation of fatty acid, while leptin takes part in abnormal conditions such as obesity and insulin resistance [43,44,45,46]. Thus, our observation that JNJ greatly lowers the mRNA expression levels of leptin, but not adiponectin, further supports JNJ as a novel therapeutic for the obesity remedy and its related diseases linked with leptin overexpression.

AMPK is a heterotrimeric protein complex comprising α, β, and γ subunits, which take a specific role in stability and regulation [47]. It is essential in energy metabolism and balance [48]. AMPK is activated through the T172 phosphorylation site within the α subunit, and AMPK activation results in adipogenesis inhibition [49,50]. AMPK activation also inhibits anabolic processes that consume ATP while activating catabolic processes that compose ATP [51], partly by controlling metabolic enzyme phosphorylation, including ACC [52]. AMPK activation instigates ACC phosphorylation on S79, which interferes with the ability of ACC to stimulate fatty acid synthesis [53]. Of interest, here, we demonstrate that JNJ upregulated AMPK phosphorylation, but downregulated ACC expression and phosphorylation during the adipocyte differentiation of 3T3-L1 preadipocytes. These findings propose that JNJ’s lipid-lowering effect is also owing to the AMPK activation and ACC reduction that could inhibit anabolic processes which consume ATP, such as fatty acid synthesis. It has been reported that LKB-1 activity and the cellular AMP/ATP ratio changes lead to induce AMPK phosphorylation (activation) on T172 [22,23]. Assuming the present findings that JNJ has abilities to elevate LKB-1 phosphorylation and reduce ATP levels during 3T3-L1 preadipocyte differentiation, it is conceivable that the JNJ-induced AMPK activation during the cell differentiation is closely linked to LKB-1 activation and decreased ATP content. In addition, considering that AMPK activation inhibits adipogenesis, the JNJ’s anti-adipogenic effect seen herein is further likely to be due to the drug-induced AMPK activation.

In addition to JNJ, we utilized c-Met gene silencing to affirm the role of c-Met in fat accumulation during adipogenesis of 3T3-L1 cells. Importantly, we showed that the knockdown of c-Met leads to less fat accumulation and TG levels throughout 3T3-L1 preadipocyte differentiation. These findings indicate that the expression and activation of c-Met are essential for fat accumulation during adipogenesis of 3T3-L1 cells. Moreover, considering that c-Met knockdown further leads to the reduction in C/EBP-α, PPAR-γ, FAS, ACC, and perilipin A during adipogenesis of 3T3-L1 cells, It appears that c-Met acts as an upstream regulator of C/EBP-α, PPAR-γ, FAS, ACC, and perilipin A during the adipogenic process, and that the lipid-reducing effect by c-Met knockdown in 3T3-L1 preadipocyte differentiation was further due to downregulation of these adipogenesis/lipogenesis/fat storage-related markers. Compared to previous studies that capmatinib, another c-Met inhibitor, attenuates adipogenesis/lipogenesis, which is mediated via AMPK phosphorylation and the reduced expression of SREBP1, C/EBP-α, FAS, and SCID1 [32] and miR-206-3p inhibits adipogenesis by blocking c-Met expression, which leads to inhibition of the phosphorylation of its downstream signal mediator Akt [33], the novelty of the present study is likely to be that the lipid-lowering effect by JNJ or c-Met knockdown during adipogenesis of 3T3-L1 cells is through alterations of the expression and phosphorylation levels of C/EBP-α, PPAR-γ, FAS, ACC, perilipin A, AMPK, and LKB-1, and ATP content.

In conclusion, this is the first study to show that c-Met is highly expressed and phosphorylated during the adipocyte differentiation of 3T3-L1 preadipocytes and that gene silencing of c-Met or pharmacological inhibition of c-Met by JNJ leads to a strong reduction in lipid accumulation during the preadipocyte differentiation. The lipid-reducing mechanism during the 3T3-L1 preadipocyte differentiation by c-Met knockdown or JNJ is mediated via control of the intracellular expression and phosphorylation levels of C/EBP-α, PPAR-γ, FAS, ACC, perilipin A, AMPK, and LKB-1 as well as ATP content. This work advocates c-Met and JNJ as potential novel targets for new anti-obesity drugs.

## 4. Materials and Methods

### 4.1. Materials

JNJ38877605 (JNJ) was obtained from Selleckchem (Houston, TX, USA). Primary antibodies for anti-C/EBP-α, anti-PPAR-γ, anti-phospho (p)-STAT-3 (Y705), and anti-STAT-3 also secondary goat anti-Rabbit and goat anti-Mouse IgG antibodies were purchased from Santa Cruz Biotechnology (Delaware, CA, USA). Anti-p-AMPK (T172), anti-AMPK, anti-p-ACC (S79), and anti-ACC antibodies were ordered from Cell Signaling Technology (Danvers, MA, USA). Anti-perilipin A antibody was bought from Bio Vision (Milpitas, CA, USA). Anti-FAS antibody was from BD Bioscience (San Jose, CA, USA). Anti-β-actin antibody, 3-isobutyl-1-methylxanthine (IBMX), dexamethasone, and insulin were acquired from Sigma (St. Louis, MO, USA).

### 4.2. Cell Culture and Differentiation

3T3-L1 preadipocyte (ATCC, Manassas, VA, USA) were grown in DMEM containing 10% heat-inactivated fetal calf (FCS) (Gibco, Grand Island, NY, USA) and 1% penicillin/streptomycin (Welgene, Daegu, Korea). To induce 3T3-L1 differentiation, DMEM was supplemented with 10% heat-inactivated fetal bovine serum (FBS) (Welgene) with the addition of a cocktail of hormones (MDI): 0.5 mM IBMX (M), 0.5 µM dexamethasone (D), and 5 µg/mL insulin (I). Consequently, the differentiation medium was replaced after 48 h with Dulbecco’s Modified Eagle’s Medium (DMEM) supplemented with 10% FBS and 5 µg/mL insulin for further 3 days. The cells were nourished every other day with DMEM containing 10% FBS until day 8. In certain experiments, the media was also added at all time points with various concentrations (5, 10, and 20 µM) of JNJ.

### 4.3. Stable Transfection of Short-Hairpin RNA (shRNA) of c-Met

3T3-L1 preadipocytes seeded into 6-well plates were transfected for 6 h with control or c-Met shRNA (Santa Cruz Biotechnology, Delaware, CA, USA) using Lipofectamine^TM^ 2000 (Invitrogen, Waltham, MA, USA). The shRNA plasmids also contain a puromycin resistance cassette. In the transfected cells, the culture medium was taken out and refreshed with DMEM containing 10% FCS, followed by 48 h incubation. The transfected cells were then selected for plasmid expression in DMEM containing 1 μg/mL puromycin for two weeks. The differentiation of stably transfected 3T3-L1 cells harboring control or c-Met shRNA was induced as above.

### 4.4. Oil Red O Staining

Oil Red O working solution was composed by mixing 6 mL of 0.1% Oil Red O in isopropanol and 4 mL of double-distilled water. On the differentiation day 8, differentiated 3T3-L1 cells were washed with PBS, fixed with 10% formaldehyde for 2 h at room temperature (RT), washed with 60% isopropanol, and dried. The fixed cells were stained with Oil Red O working solution (Sigma, St. Louis, MO, USA) for 1 h at RT and washed with distilled water. Lipid droplets were then visualized under light microscopy (Nikon, TS100, Tokyo, Japan).

### 4.5. Analysis of Cell Counting

3T3-L1 preadipocytes were seeded in 24-well plates and differentiated as explained above. On day 8 of differentiation, cells were stained with trypan blue dye, and those that did not take up the dye were counted under a light microscope.

### 4.6. Intracellular Triglyceride (TG) Content Quantification by AdipoRed Assay

On day 8 of differentiation, intracellular TG contents were quantified using the AdipoRed Assay Reagent kit in accordance with the manufacturer’s instructions (Lonza, Basel, Switzerland). Fluorescence was defined after a 10 min incubation on Victor3 (Perkin Elmer, Waltham, MA, USA) with excitation at 485 nm and emission at 572 nm.

### 4.7. Whole-Cell Lysates Preparation

3T3-L1 cells were washed with PBS and lysed in a modified radioimmunoprecipitation assay buffer (Sigma) at appointed time points. The cell lysates were extracted and centrifuged at 12,000 rpm for 15 min at 4 °C, and the supernatant protein concentration was concluded using the bicinchoninic acid assay kit (Thermo Fisher Scientific, Rockford, IL, USA).

### 4.8. Immunoblotting Analysis

The separation of protein was done by SDS-PAGE electrophoresis and moved onto nitrocellulose membranes (Millipore, Bedford, MA, USA). The membranes were washed with Tris-buffered saline (TBS) (10 mM Tris-Cl, 150 mM NaCl, pH 7.5) containing 0.05% (*v/v*) Tween 20 (TBST) followed by blocking in TBST supplemented with 5% (*w/v*) non-fat dried milk. The membranes were incubated overnight with certain primary antibodies at 4 °C. The membranes were then rinsed with TBST and further incubated with an HRP-conjugated secondary antibody (anti-goat IgG, anti-mouse IgG, or anti-rabbit IgG) for 2 h at RT. Membranes were then rinsed three times with TBST and proceeded with enhanced chemiluminescent reagents. β-Actin expression levels were utilized as a control for uniform protein loading.

### 4.9. Reverse Transcription-Polymerase Chain Reaction (RT-PCR)

Total cellular RNA was isolated with RNAiso Plus (TaKaRa, Kusatsu, Shiga, Japan). As previously described, three micrograms of total RNA were converted using a random hexadeoxynucleotide primer and reverse transcriptase [53]. Single-stranded complementary DNA was then amplified by the PCR with the following primers: C/EBP-α sense 5′-TTACAACAGGCCAGGTTTCC-3′; antisense 5′-CTCTGGGATGGATCGATTGT-3′; PPAR-γ sense 5′-GGTGAAACTCTGGGAGATTC-3′; antisense 5′-CAACCATTGGGTCAGCTCTC-3′; FAS sense 5′-TTGCTGGCACTACAGAATGC-3′; antisense 5′-AACAGCCTCAGAGCGACAAT-3′; perilipin A sense 5′- CTTTCTCGACACACC ATGGAAACC -3′; antisense 5′- CCACGTTATCCGTAACACCCTTCA -3′; leptin sense 5′-CCAAAACCCTCATCAAGACC-3′; antisense 5′-CTCAAAGCCACCACCTCTGT-3′; adiponectin sense 5′-GGAGATGCAGGTCTTCTTGGT-3′; antisense 5′-TCCTGATACTGGTCGTAGGTGAA-3′; actin sense 5′-GGTGAAGGTCGGTGTGAACG-3′; antisense 5′-GGTAGGAACACGGAAGGCCA-3′. β-actin mRNA expression levels were utilized as an internal control.

### 4.10. Intracellular ATP Levels Measurement

3T3-L1 preadipocytes were seeded in 96-well plates and grown in the presence of differentiation media with or without JNJ or 2-deoxyglucose (2-DG). On days 2, 5, and 8 of differentiation, intracellular ATP levels were quantified by luciferase activity as explained by the manufacturer’s protocol (ATPLite-1step, PerkinElmer, CT, USA). After a 2 min incubation, luminescence was assessed on a Victor3 (PerkinElmer).

### 4.11. Statistical Analysis

Cell count analysis was carried out in triplicates and repeated three times. Data are expressed as mean ± standard error (SE). One-way ANOVA followed by Dunnett’s post hoc test was done using SPSS 11.5 software (SPSS Inc., Chicago, IL, USA). *p* < 0.05 was contemplated to indicate statistically significant differences.

## Data Availability

The data that support the findings of this study are available from the corresponding author upon reasonable request.

## Supplementary Materials

The supporting information can be downloaded at: https://www.mdpi.com/article/10.3390/ijms24098086/s1. Figure S1: INCB28060, another c-Met inhibitor, reduces lipid accumulation and TG content during 3T3-L1 preadipocyte differentiation.
