# Supplementary material for: The Receptor Tyrosine Kinase c-Met Promotes Lipid Accumulation in 3T3-L1 Adipocytes
Source: Int J Mol Sci. 2023 Apr 29;24(9):8086. doi: 10.3390/ijms24098086 (PMC10179087; doi:10.3390/ijms24098086)
Supplement: Supplementary file 1 [file ijms-24-08086-s001.zip › ijms-2342611-supplementary.pdf]

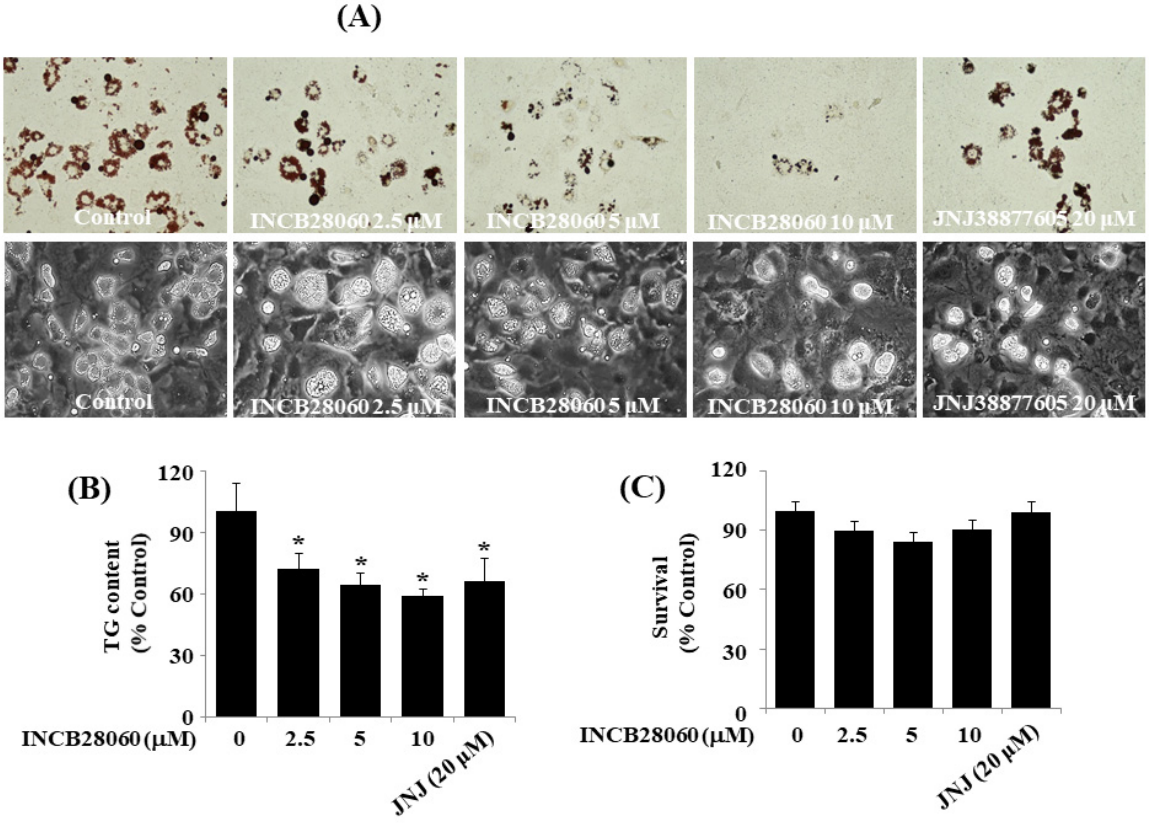

Figure S1. INCB28060, another c-Met inhibitor, reduces lipid accumulation and TG content during 3T3-L1 preadipocyte differentiation.
